# What is new in the early health technology assessment’s new definition?

**DOI:** 10.1017/S0266462326103821

**Published:** 2026-05-21

**Authors:** Manuel Antonio Espinoza

**Affiliations:** School of Public Health, Li ka Shing Faculty of Medicine, The University of Hong Kong, Hong Kong (SAR), China

**Keywords:** health technology assessment, value, decision making, early assessment, cost-effectiveness, research and development, innovation

## Abstract

Early health technology assessment (HTA) has recently been defined as a process for evaluating the “potential value” of technologies at early development stages. This analysis examines how value is characterized in early stages compared to standard HTA. Using the MAPS framework (Methods, Attributes, Procedures, and Social Preferences), the findings indicate that a broader value framework may be applicable to early HTA relative to standard HTA. This framework includes systematic consideration of attributes beyond health-related benefits, such as real option value and innovation, as well as additional methods like fiscal impact analysis and social return on investment. Additionally, there is potential for less variation in early HTA processes globally, given that these assessments are less tied to local funding decisions. Finally, the role of social preferences in early HTA may also involve considering trade-offs and impact modifiers that are particularly relevant in initial stages, and which deserve further research.

## Background

Health technology assessment (HTA) in earlier stages of a technology life cycle has grown in interest across the globe over the last few years. One of the main motivations is to connect research and development (R&D) of new technologies with the subsequent decisions that determine access and utilization in health systems. The recent publication by Grutters et al. (1), which defines Early HTA, is an important contribution to the field because it promotes an area of specialization fostering continuous development.

According to Grutters et al. Early HTA is a “Health Technology Assessment conducted to inform decisions about subsequent development, research, and/or investment by explicitly evaluating the potential value of a conceptual or actual technology.” The definition relies on the value characterization, which is consistent with previous definitions (2). Likewise, as the HTA definition (3), Early HTA remains broad regarding its explanation about the meaning of value, referring to a general description of dimensions, but leaving local policy makers the responsibility of defining a value framework that responds to their own contextual needs.

However, the discussion around a new definition of early HTA offers an opportunity to revise the value characterization of technologies. For example, in early stages, we may consider certain attributes more relevant than in standard HTA. The aim of this analysis is to explore whether the idea of “potential value” included in the new definition of early HTA is the same as in standard HTA or may have some specific characteristics. To facilitate a structured exploration, I introduce a conceptual tool called MAPS, which will be used to examine how to characterize value through a structured approach based on four pillars.

## The MAPS framework to explore alternative value frameworks

The MAPS is a straightforward conceptual tool to facilitate the analysis of a value framework. MAPS is built upon four pillars that shape how value can be characterized. They are: **M**ethods, **A**ttributes, **P**rocedures, and **S**ocial Preferences. First, methods correspond to the set of techniques and tools used to measure each attribute of value, and how their measurements should be combined to produce one common metric of value. Second, attributes are the components of the overall value, where each captures a partial share of benefits or gains attributable to the technology. Although harms or losses cannot be considered attributes of value, they may be considered in a value framework, as long as they are expressed as benefits lost and included in a measure of net value. For example, adverse effects attributable to one technology produce health losses, which must be discounted from the gains.

Third, procedures refer to the organized mechanisms and stages of the process to identify, measure, and value each attribute and its integration to produce an overall characterization of value. Procedures guide what must be done in each stage, how much time is available, and who participates, providing perspectives and insights. Hence, although attributes and methods are essential determinants of value, different procedures will also determine different outputs and should not be underestimated (4).

Finally, social preferences refer to two main elements: first, social considerations that modify the value of one or more attributes (i.e., effect modifiers or interaction variables). For example, the severity of the disease can affect the value of the benefit gained (e.g., one additional year lived may be worth more in severe than in nonsevere conditions) (5). Second, social preferences constitute the main basis for eliciting trade-offs among attributes needed to inform resource allocation decisions. For example, the trade-off between maximizing health versus achieving a more equal distribution across subgroups defined by some equity concern (i.e., equity-efficiency trade-off), which has been estimated in several jurisdictions as indices of inequality aversion (6;7), is essentially determined by social preferences.

## Standard HTA versus early HTA

### Methods

First, the new definition relies on standard methods to evaluate clinical and economic evidence. For example, it refers to the use of classical cost-effectiveness methods to identify key parameters in the value characterization. However, early HTA may also be focused on other economic approaches, such as cost–benefit analysis (8), fiscal impact analysis (9), and the social return on investment (ROI) (10), because they address different questions. For instance, additional investments can be justified in terms of maintaining leadership in certain areas of medical research or increasing productivity. These arguments may be better accepted for early HTA than standard HTA. On the other hand, in early HTA, health benefits calculated through mathematical modeling based on prior real-world data, and at times, strong assumptions may be acceptable; which contrasts with standard HTA, where estimates from clinical trials have some superiority. Further, the standard approach to characterize second-order uncertainty focused on the consequences of health production only (11) may not fit with a broader perspective of the R&D at early stages, which may also include some other economic outcomes and spillover effects (12). Indeed, these other factors may justify further investments alongside the R&D process, even though the prospects may be projected as not cost-effective.

### Attributes

Attributes are probably the core of any HTA value framework. Standard HTA often starts considering clinical benefits gained at the individual level. Then, it moves to the consideration of individual health gains through an estimate of the long-term effects on quantity and health-related quality of life (e.g., as QALYs). A second important attribute is the health gains net of the healthcare opportunity cost, often informed by cost-effectiveness analysis (13). A third set of attributes includes other social concerns, such as the impact on the distribution of health outcomes across disadvantaged and advantaged subgroups (i.e., equity), financial protection, and the health system’s responsiveness (14). Although they have been widely recognized as an important part of the process, the extent to which they are systematically considered in HTA varies largely across jurisdictions.

Among these various social concerns, several attributes attract interest because they capture some share of social welfare that is beyond health. Innovation is one of those attributes whose marginal contribution to overall value is partially explained by the health consequences, but also due to other nonhealth-related benefits. For example, innovation may promote investment and economic growth, leading to higher productivity and greater work opportunities. These attributes may be more relevant in early HTA than standard HTA, especially if the investment is supported by a public or private funder whose objective is to promote economic activity through innovation (15).

Real option value (ROV), which captures the benefit of a technology that enables patients to access future, more effective alternatives that would otherwise be unavailable, is another attribute that might be particularly relevant for early HTA (16). ROV takes special importance because the assessment in early stages should consider the broader pipeline of innovation, which can include other technologies that will be available in the future.

Another attribute is the new biological mechanism, which finds a special application in new antimicrobials (17). Although a new mechanism may be equally effective compared to another new antimicrobial that belongs to a well-known class, its value is not limited to its effectiveness against current pathogens. Instead, new mechanisms enhance the range of alternatives available to the health system, as it has the potential to address emerging multiresistant microorganisms efficaciously. Accordingly, its value is partly determined by both the health benefits achieved and the mitigation of potential economic and social crises resulting from novel infectious agents, as demonstrated during the COVID-19 pandemic (18). Finally, other attributes like the value of hope (19), insurance value (20), and value of knowing (21) can also be considered more relevant in earlier stages because they are consistent with broader goals of R&D.

### Procedures

Procedures vary largely across jurisdictions in standard HTA. In general, more robust and mature HTA processes are observed in places where HTA outputs determine the allocation and use of the health system resources. While HTA is seen as a signal of institutional strength in some countries, local decision makers may feel HTA risky in others, because it implies delegating power to a technical body that may jeopardize the budget control (22). Both views coexist and can explain the variation of procedures across jurisdictions.

However, the definition of procedures may be different for early HTA, which may achieve a higher level of consensus. In early stages, where HTA does not intervene in the health system’s resource allocation, the systematic consideration of different stakeholders’ views may find broader agreement. Indeed, their inputs may impact the R&D pipeline, as well as the investment decisions. Thus, procedures for early HTA are an area that deserves further attention.

### Social preferences

In standard HTA, social preferences are frequently incorporated into deliberative processes. For instance, determining the value of an additional QALY in children versus adults, or for severe compared to less severe diseases, often involves considerations that extend beyond empirical data. In early HTA, social preferences may involve distinct value-modifiers and trade-offs. For example, if ROI is considered relevant in early HTA, innovation might serve as a key modifier. Thus, each dollar of ROI could be worth differently depending on whether it results from investment in an antimicrobial with a new mechanism of action or from a “same-class” antibiotic, even when both demonstrate equivalent effectiveness against current pathogens.

Alternatively, it may be necessary to consider trade-offs when allocating resources among different investment options. For example, a country might choose to invest in higher-risk opportunities that are considered innovative, even if their returns are lower compared to alternative investments in less risky portfolios, such as established technologies. In standard HTA, social preferences generally focus on the health outcomes of accepting or rejecting new technologies, whereas in early HTA, social preferences may also reflect broader socioeconomic aims, including economic growth, research innovation, and scientific leadership.

Table 1 presents a synthesis of the comparative analysis between standard HTA and early HTA, examined through the lens of the MAPS framework.

**Table 1.** Comparative analysis of the value characterization in standard HTA versus early HTA using the MAPS framework Table 1. long description.

	Standard HTA	Early HTA
Methods	Health: Evidence-based medicine Economic: Cost-effectiveness analysis; budget impact analysis Uncertainty: Second-order uncertainty of clinical treatment effects and cost-effectiveness estimates	Health: Health modeling Economic: Cost-effectiveness, Return on investment, cost–benefit, and fiscal-benefit analysis Uncertainty: It may include second-order uncertainty about other economic consequences, such as impact on productivity gains, labor, and spillover effects Social: Additional methods are required to systematically characterize certain attributes of social value
Attributes	Health: Clinical benefits, safety, impact on burden of disease, and population health Economic: Net benefits. Social, cultural, and ethical issues are often broadly mentioned, and their application varies largely across contexts. They may include equity, end-of-life considerations, social vulnerability, and rules of rescue	Health: clinical benefits, safety, impact on burden of disease, and population health Economic: Net benefits, Return on investment, cost–benefit, and fiscal-benefit analysis Social: Innovation, real option value, novel action, value of hope, and spillover effects
Procedures	The relevance, consistency, and reliability of procedures depend on the extent to which health systems follow HTA outputs. In general, more systematic and robust procedures are seen in jurisdictions where governments rely on HTA to support their decisions about resource allocation. This willingness to follow the HTA process leads to wide procedural variations across jurisdictions	Because early HTA outputs are less connected with allocation of resources in the health system than standard HTA, there is an opportunity to generate broader consensus about procedures for early HTA
Social preferences	Social preferences often take place in deliberative stages. In these contexts, different stakeholders shared their views about how the value of certain estimates (e.g., expected health gains) may change due to certain social considerations that matter. Furthermore, they also discuss the trade-offs society may be willing to make in order to satisfy other objectives, for example, equity efficiency trade-offs. In standard HTA, social preferences are more related to the direct consequences of adopting or rejecting new technologies for people in need	Social preferences in early stages may want to capture broader economic and social considerations. Although modifiers and trade-offs can still focus on populations in need and their direct health consequences, it may also consider factors like the effect of innovation on other goals, like economic growth, research innovation, and scientific leadership

## Conclusions

The revised definition of early Health Technology Assessment (HTA) highlights two fundamental aspects: the objective of supporting decisions at the initial phases of the technology life cycle, and the instrumental function of assessing “potential value.” The analysis of value characteristics in the early HTA context presented in this manuscript suggests that “potential value” possesses distinct attributes compared to conventional HTA. Furthermore, value frameworks developed for early HTA may enable wider international agreement, as they are generally less pivotal for local coverage determinations. Continued efforts to develop comprehensive value frameworks for early HTA would represent an important advancement in the discipline and merit further research.

## Table 1. Long description

Starting from the top row, the Methods row compares Standard H T A, which uses evidence-based medicine, cost-effectiveness, budget impact analysis, and considers second-order uncertainty in clinical and economic outcomes, with Early H T A, which includes health modeling, cost-effectiveness with adjustments, return on investment, cost-benefit, fiscal-benefit analysis, and broader uncertainty including productivity, labor, spillover effects, and social value characterization. The Attributes row shows both approaches address clinical benefits, safety, disease burden, and population health, but Early H T A adds return on investment, cost-benefit, fiscal-benefit, innovation, real option value, novel action, value of hope, and spillover effects, while Standard H T A broadly mentions social, cultural, ethical issues such as equity, end-of-life, vulnerability, and rules of rescue. The Procedures row notes Standard H T A procedures depend on health system reliance and vary across jurisdictions, while Early H T A offers opportunities for broader consensus due to less direct resource allocation linkage. The Social Preferences row highlights that Standard H T A focuses on direct consequences for populations in need and deliberative stakeholder input, whereas Early H T A aims to capture broader economic and social considerations, including innovation’s impact on economic growth, research, and scientific leadership.

Navigate back to Table 1..
